# Umbilical Cord Tissue-Derived Cells as Therapeutic Agents

**DOI:** 10.1155/2015/150609

**Published:** 2015-07-12

**Authors:** Olga Maslova, Miroslav Novak, Peter Kruzliak

**Affiliations:** ^1^National Taras Shevchenko University of Kyiv, Kyiv, Ukraine; ^2^Hemafund Ltd., Lisozakhysna Street 5, Kyiv, Ukraine; ^3^International Clinical Research Center, St. Anne's University Hospital and Masaryk University, Pekarska 53, 656 91 Brno, Czech Republic

## Abstract

Although the characteristics of SC, including UC-derived cells, are a dramatically discussed issue, this review will focus particularly on some controversial issues regarding clinical utility of cells isolated from UC tissue. UC-derived cells have several advantages compared to other types and sources of stem cells. The impact of UC topography on cell characteristics is briefly discussed. The necessity to adapt existing methods of cell isolation and culturing to GMP conditions is mentioned, as well as possible cryopreservation of this material. Light is shed on some future perspectives for UC-derived cells.

## 1. Introduction

There is a plethora of papers covering the topic of stem cells (SC) and their potential for use in regenerative medicine [1–5]. To date various types of stem cells have been described in humans from a variety of tissues, including preimplantation embryos, foetuses, birth-associated tissues, and different adult tissues [6]. Based on biochemical and genomic markers, they can be broadly classified into embryonic stem cells (ESC), mesenchymal stem cells (MSCs), and haematopoietic stem cells (HPS). The so-called neonatal MSC sources, including the placenta, amniotic fluid, and UC, have fewer limitations than cells from other tissues. It has been shown that the cells in these organs are more similar to early embryonic cells, both in surface marker portrait and differentiation potential. The UC is rich in cell material and is the most homogeneous formation in comparison with other provisional organs [7].

One of the most promising sources of SC, UC tissue, has been discussed in different reviews and research papers. UC-derived cells have been under thorough investigation since 1991 [8] and the view on their biology has been developing intensively [9–15]. Hundreds of clinical trials are currently carried out using cells obtained from UC tissue. Moreover, cord tissue is considered a commercialized product for cryobanking on a par with cord blood (CB) in some countries [16, 17]. This cell population is mentioned as a source of cell material for usage in various fields of regenerative medicine [18, 19]. Human UC is a rich source of stem and progenitor cells (MSCs) derived either from the cord tissue or from cord blood [20]. However, CB is mostly considered the source of haematopoietic stem cells (HSC) [21] and UC can be considered a better source of MSC [22]. Usually the cells obtained from UC tissue are referred to as mesenchymal stem cells or multipotent stromal cells, both abbreviated as MSCs. They completely meet the classical criteria for MSCs: plastic adhesion, positive marker expression (CD105, CD90, and CD73), and trilineage differentiation capacity [23, 24]. However, it has been shown in a number of works that these cell populations exhibit broader “stem features” than MSCs from adult sources [25, 26]. Taking into account that the UC itself is far more available and ethically “clean” than other described SC sources, it becomes obvious that UC could be called a “stem cell goldmine.”

Several excellent reviews focused on the characteristics of UC cells and clinical research are currently available. For example, the work of Kim et al. [27] describes in detail the main properties of UC-derived cells that allow them to be used in regenerative medicine. Moreover, this review provides very useful data on WJ-MSCs as therapeutic agents for different pathologies. Prasanna and Jahnavi [28] prepared a comprehensive review of the data regarding the regenerative and immunomodulatory characteristics of WJ-MSCs. Bongso and Fong [29] carried out an in-depth analysis of the challenges and future clinical directions in relation to UC-derived cells. Nagamura-Inoue and He [30] summarized concisely the advantages and potential clinical utility of UC-derived cells. All these reviews provide sufficient information on the ontogenesis of UC and properties of UC-derived cells such as surface marker expression, differentiation capacities, and paracrine potential. It must be mentioned that the differentiation capacities of UC-derived cells are significantly higher than originally thought when MSC research began, because every year there are new works on successful novel cell-type differentiation from UC-derived cells [31–33]. For example, one of the new papers is “Epimorphin-Induced Differentiation of Human UC Mesenchymal Stem Cells into Sweat Gland Cells” [34].

In order to avoid broad overlaps and repetition of information, it is planned that this paper will focus on some controversial issues.

## 2. Topical Issues Related to Utility of UC-Derived Cells in Regenerative Medicine

### 2.1. The Impact of UC Topography on Cell Characteristics

Unlike the adult organism, where mesenchyme is completely transformed into a variety of connective tissues, the UC, as a yolk sac and allantois derivative, contains the primitive form of extraembryonic mesenchyme. The cells in the UC are divided into different groups based either on the region of isolation (WJ, cord lining (CL), perivascular area (PA), etc.) or on the cell type (epithelial, stromal, smooth muscle, and endothelial cells) [69–71]. A range of authors describe differences in the morphofunctional characteristics of cells isolated from different anatomic areas of the UD (e.g., WJ, PA, CL, and vascular walls) [72–74]. However, the majority of papers that investigate and describe UC cells* in vivo* (in both animals and humans) are based on the use of accumulated fraction of cell material isolated from the whole UC tissue or WJ [75–78]. This is mainly due to the simplicity of isolating mesenchymal cells from the whole UC tissue, precluding the necessity for additional operations. Moreover, Mennan et al. [79] showed that cells from whole UC differentiated as, or better than, those isolated from individual cord regions and therefore have potential as a useful source for obtaining promising cell populations for further study. In their study, MSCs from four regions of the same cord (artery, vein, WJ, and CL), in addition to a mixed cell population from the whole cord, were isolated and compared for potential musculoskeletal cell therapy. MSCs were cultured from all individual cord regions, as well as enzymatically digested whole cord, demonstrated by their plastic adherence, flow cytometry profile, and ability to differentiate along osteogenic, adipogenic, and chondrogenic lineages. The growth kinetics and MSC immunoprofile showed no significant difference between cells from any of the populations (or isolates). Osteogenic and adipogenic differentiation studies showed variations between cord regions, with the best differentiation seen in WJ and whole cord. Chondrogenic differentiation showed little difference between cells isolated from different cord regions. Unfortunately there is not enough evidence for priority of any of the cord regions for clinical utility. It could be assumed that the most effective strategy nowadays is to use WJ for cells' isolation.

Nevertheless, undoubtedly, the research of mesenchymal cell subpopulations is still an important task for fundamental cell biology. The investigation of UC stem cell expression of known markers of the embryonic stage, such as Oct-4 and SSEA4, is also of special interest. A variety of authors introduced data relating to the expression of these markers by cells isolated from the UC [73, 80–82]. This was one of the main factors that demonstrated the special position of UC-derived SC and allowed the suggestion that the properties of these cells, in the ontogenetic sense, are closer to those of pluripotent embryonic cells than to those of adult multipotent cells. At the same time, there is much evidence of revaluation of expression of these markers in UC-derived cells. For instance, there were presumably some methodological inaccuracies concerning the level of expression of Oct-4. As summarized in Ryan et al. [83], critical examination of the Oct-4 literature prompts the suggestion that Oct-4 expression in foetal MSC may be a case of “The Emperor's New Clothes,” with early reports of (false) positive expression amplified in subsequent studies without critical attention to emerging refinements in knowledge and methodology. In turn, the role of SSEA4 could be another, for there is some new evidence of changes in the viewpoint regarding the influence of SSEA4 on proliferation of stem cells. The paper by He et al. [84] reveals that SSEA4 may display altered expression profiles in response to culture media containing FBS and may not be an essential marker of WJ-MSC pluripotency. It should be mentioned that expression of early embryonic transcript markers is not well correlated with the differentiation potential of MSC. In order to link the expression of these markers and differentiation abilities, relevant and scientifically sound evident should be enclosed. Various differentiation paths should be analyzed by morphological, biochemical, and most importantly the functional studies and experimentally supported.

It must be understood that the data on expression of markers by UC cells vary in different laboratories. It is possible that during these investigations the authors faced not only different methods but also heterogeneity of UC samples due to the individual features of the donors, including ethnic group differences and other unrevealed characteristics [85]. This issue is yet to be investigated, but the distinction of results achieved by different authors validates this explanation.

However, despite the necessity for more in-depth research of the molecular biological peculiarities of the functioning of different subpopulations of UC tissue, the efficiency of their use as therapeutic agents seems obvious. Thus, hundreds of clinical research projects using UC-derived cells have been registered to date, and there are even more preclinical research projects. For example, 283 studies can be found for “cord tissue” at https://clinicaltrials.gov/. Entering the search request “umbilical cord tissue cell clinical trial” at http://scholar.google.com/ leads to 44,500 results including patents and excluding citations. The variety of pathologies that assume using UC-derived cells is impressive.

### 2.2. Possible Mechanisms of Action

UC-MSC not only can be used in the direct cell-oriented way as a suspension but also can produce different chemofactors in a culture medium that could be used either as a conditioned medium for culturing other cell types or as a potential therapeutic substance [86, 87]. It has been demonstrated that MSC culturing on WJ extract delays senescence through p53 and p16INK4a/pRb pathways [88]. The authors of this research suggest that WJ extract provided an ideal microenvironment for MSC culture expansion* in vitro*, preserved MSC properties by delaying MSC senescence, and allowed large numbers of MSCs to be obtained for basic research and clinical therapy. Additionally, these MSCs may become a kind of supportive environment for some other cell types. Stromal support for HSCs [89], spermatogonial SC, and ESC was observed [90]. According to Fan et al. [91], mesenchymal stromal cell supported UC blood* ex vivo* expansion, enhances regulatory T cells, and reduces graft versus host disease. Moreover, Lin et al. [92] have shown that the post-UC-cell medium enhances freeze-thaw survival and expansion of cryopreserved CD34+ cells.

Paracrine effects of WJ-MSC could be performed through multiple signalling pathways with different key molecules. These effects provide neuroprotection, angiogenesis, and enhanced regeneration. Functional validation showed that WJ-MSCs induced better neural differentiation and neural cell migration via a paracrine mechanism. It has been confirmed that NTF3, EGF, MDK, HBEGF, CXCL2, CXCL5, and FGF9 are more abundant in WJ-MSCs than in MSC from other sources [56]. As it has been demonstrated in [66], “human WJ-MSCs upregulated the mRNA transcript expression of TGF-*β*2, hypoxia-inducible factor-1*α*, and plasminogen activator inhibitor-1 genes in normal skin fibroblasts in our culture conditions. Other genes involved in re-epithelialization, neovascularization and/or remodeling – including VEGF, fibroblast growth factor-2, connective tissue growth factor, collagen I and collagen III – were not changed. Decorin and TGF-*β*3 also remained unaffected.” Nascimento et al. [43] have concluded that the expression of angiogenesis-associated transcripts (subtypes of VEGF, angiopoietins, HGF, C-mET, bFGF, TGF-*β*, and PDGF-AB) in cord-derived cells is high. Therefore, several factors are most likely contributing for the cardioprotective effects observed* in vivo* and* in vitro*. More-directed work is already under way specifically to identify the role of each of the candidates. The same situation is with other effects of MSC: the possible candidate molecules are discussed but the precise mechanisms and signal pathways are not elucidated enough.

WJ-MSC-based therapy can be considered as a potential alternative to orthotopic liver transplantation for liver disease treatment. UC-derived cells have demonstrated a potential to differentiate into hepatocyte-like cells. The* in vitro* and* in vivo* use of UC-MSCs for liver cell therapy has been described [93]. This cell population displays some of hepatic markers characterizing the sequential steps of liver development. In treating liver cirrhosis, UC-MSCs act like anti-inflammatory and antifibrosis agents by endogenous secreted metalloproteinases [27]. Potential treatment of cardiovascular diseases using WJ-derived cells is described [94]. Surgical treatment using nonautologous valves or conduits has many disadvantages, including obstructive tissue ingrowths and calcification of the implant, and consequently cardiovascular foetal tissue engineering focuses on the* in vitro* fabrication of autologous, living tissue with the potential to regenerate heart muscle [27]. WJ-derived cells are a promising cellular source for cartilage repair due to both their differentiation and immunomodulatory properties [27]. WJ-MSCs have been demonstrated to successfully differentiate into cells resembling mature chondrocytes. Their peculiar features of low immunogenicity and the potential to induce immune tolerance justify efforts towards the use of UC-derived cells in osteoarthritis, rheumatoid arthritis, and other disease settings. Recent tissue engineering studies have focused on the development of bioartificial nerve conduits to guide axonal regrowth. Given the intrinsic ability of activated Schwann cells to promote axonal regeneration* in vivo*, UC-MSC can successfully be used to derive mature Schwann cells for the regeneration of peripheral nerves. Schwann cells also support axonal regeneration, construct myelin, and contribute to functional recovery in a spinal cord injury model [27]. Moreover, it has been established that UC-derived cells could be of great interest in human perinatal disorders of the central nervous system. The influence on the immune system and the inflammation process is also widely discussed [95–97]. Another interesting question is the potential use of UC-derived cells for anticancer therapy [98, 99]. Today there is evidence of the potential influence of UC-derived cells on almost every physiological system (Table 1).

The healing potential of UC-derived cells, as well as other MSCs, in regenerative medicine could be associated with different mechanisms such as direct reparation and tissue remodelling, paracrine effects and influence on the microenvironment, and immunomodulation. In most cases, these influences can be combined. Furthermore, methods of analysis are not always capable of checking all stages of performance of the clinical effect that is why we can only evaluate the final result—the positive or negative influence of the treatment.

## 3. Adaptation of Methods to GMP Conditions

One of the prominent tendencies in modern cell therapy is standardization of methods and adaptation of these methods to good manufacturing practice (GMP). The question of standardizing cell-isolation methods is one of the most widely discussed issues [100–103]. The paper by Sart et al. [104] gives sufficient data on this topic. It is also covered elsewhere [105, 106]. Usage of GMP-compliant conditions can make products safer for patients and researchers, but the price of cell products (that are not cheap even without such conditions) should be significantly higher.

Complying with GMPs requires precisely defining the production process(es) as well as the multiple criteria required for a quality final product. Such variables include the environment, staff training and qualification, and controls [107]. GMP-compliant processes, cell culturing and preparation to clinical use, should be performed in accordance with general requirements for pharmaceutical facilities (building architecture, air preparing and cleaning systems, special requirements to the personnel, etc.). Preference is given to xeno-free conditions of culturing. Design of new clinical-grade approaches for obtaining cell-based products from stem cell sources, including UC, is widely discussed [108–111].

As Martins et al. [112] clearly emphasize, “Due to the novelty, complexity and technical specificity of cell therapy, specially tailored and harmonized regulations were necessary to ensure global availability of cellular products. Currently, in the European Union, the regulation (EC) No. 1394/2007 on Advanced Therapy Medicinal Products (ATMPs) lays down specific guidelines concerning centralized authorization, supervision, and pharmacovigilance. One of the most important requirements of ATMPs is the full characterization of the product. Safety is a major concern with this type of biopharmaceutical.” For this reason, the authors of that work designed the ATMP with a registered UCX trademark. A method to consistently isolate, expand, and cryopreserve a well-characterized population of human UC-derived MSCs has been successfully adapted. Expression profiling, immunophenotypic analyses, mixed lymphocyte reaction, karyotyping, and evaluation of teratoma-forming potential as well as differentiation, immune suppression, and treg conversion assays were performed. Moreover, the most suitable GMP-grade cryopreservation and recovery methods are proposed for this cell-based product.

### 3.1. Cryopreservation

The safest and most effective cryopreservation method is an important part of the research connected with clinical utility of all kinds of SC. A few recent papers focus on this problem. In Roy et al. [113], a serum-free formulation of 10% dimethyl sulfoxide (DMSO) and 0.2 M sucrose for cryopreservation of UC tissue was optimized. Slow freezing and rapid thawing were adopted. MSCs harvested from WJ of cryopreserved UC could undergo robust expansion, differentiate to mesodermal lineages, and express MSC-characteristic surface antigens. The cumulative cell yield, however, was lower compared to corresponding fresh cord tissue. Other papers gave alternative methods. Di Giuseppe et al. [114] carried out profound research on the cryopreservation effects on WJSCs proteome. It was demonstrated that frozen WJSCs showed qualitative and quantitative changes compared to fresh cells, expressing proteins involved in replication, cellular defence mechanism, and metabolism, which could ensure freeze-thaw survival. The results of this study could play a key role in elucidating possible mechanisms related to maintaining active proliferation and maximal cellular plasticity, thus making the use of WJSCs in cell therapy safe following biobanking. Consequently, establishment of the cryoconditions suitable for further GMP-compliant utility is of crucial importance.

**Figure 1:**
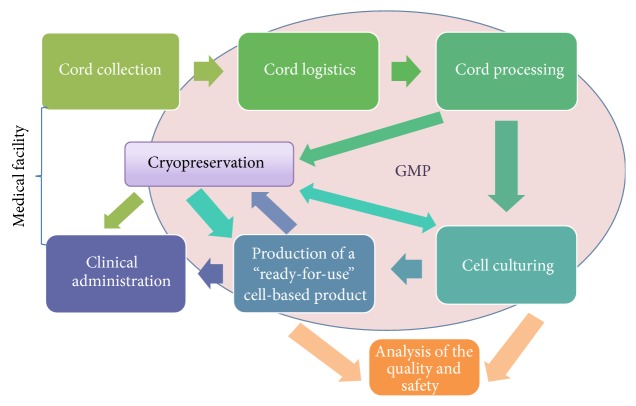
Schematic demonstration of the interconnections between the processes involved in obtaining of the product under GMP conditions.

Based on the available information we offer to depict the processes involved in obtaining the product under GMP conditions in Figure 1.

The way of umbilical cord in regenerative medicine begins and ends in medical institutions. At birth the material should be properly collected and then transported in suitable conditions to properly built and serviced gmp-grade facilities, where it should be processed for cells' isolation and culturing and preparation of medicinal product. The material might be cryopreserved at different stages: as a cord tissue, as a cell culture, or as a “ready-for-use” product. Moreover, the quality and safety studies need to be performed during manufacturing of the product. Some issues remain unresolved in the subject of choosing the necessary analyses set. What tests should the umbilical cord cell-based product pass? Apparently it depends on the source (autologous or allogeneic) of cord cells, the purpose of its use (type of pathologic condition), and so forth. Sterility and safety testing, immunophenotyping, testing of differentiation potential, karyotype analysis, telomerase activity assay, angiogenesis assays, various tumorigenicity assays, DNA-damage detecting assays, and so forth are among tests that could be established. The “ready-for-use” product is given to medical professionals and administrated in clinics.

One of the most important requirements of clinical grade cell-based products is the full characterization of the product. Safety is a major concern with this type of biopharmaceutical. The cell-based product must not cause infections, allergies, or malignancies [112]. One of the obstacles is variety of GMP-compliant conditions, because there are no strict standard requirements for production of cell-based products. It has been shown that MSCs processed under different variations of GMP-conditions could differ in their properties [115].

The most exigent part of above-mentioned interconnection is standardisation of procedures and successful cooperation between medical doctors, scientists, and pharmaceutical specialists.

## 4. Future Perspectives and Challenges

MSC in culture is a heterogeneous group of multipotent cells that are likely to acquire certain phenotypic properties after isolation from different tissues: the expression of a set of surface markers, adhesion to plastic, and the ability to induce differentiation [116]. Thus, an understanding of the cytological and biochemical specificities of MSC not only in culture but also in the living organism is a key issue that must be solved for more efficient and safer application of this material in clinical practice [117].

There are two prominent approaches towards stem cell-based therapeutics. The first can be characterized as science-oriented and the second as medicine-oriented. The science-oriented approach aims to reveal the deep molecular and cellular mechanisms of stem cell action* in vitro* and* in vivo*. The more characteristics that can be evaluated and fully described, the more features there are to analyse: here is their vector. And as there are more and more new findings and characteristics every year, presumably this analytical process could be virtually endless [118]. For example, another one-gene screening of WJ-MSCs has been performed by Mechiche Alami et al. [119]. The medicine-oriented approach focuses on the final effects; it targets the result, even without a profound understanding of the mechanisms behind the action of therapeutic agents. Thus, the latter approach is more risky.

Consequently, the most effective strategy should combine the above-mentioned methods of stem cell usage. Some main characteristics should be chosen for analysis (and they must be both informative and available for routine analysis), and then the most appropriate method of application should be selected and the therapeutic effect evaluated.

The most prominent future challenges and perspectives tend to concentrate on the field of standardization, GMP-grade optimization of all the relevant processes, and the search for procedures that minimally affect the stemness properties of cells.

## 5. Conclusions

The vast majority of UC-MSC clinical research is focused on remodelling of connective tissue injuries and repair of various organ malfunctions due to direct cell substitution or through certain paracrine interactions. UC-derived cells can be considered a mixture of cell types with broad therapeutic potential, the most distinguished of which are MSCs that possess properties combining foetal and adult SC features. Different authors have clear evidence about distinct properties of cells isolated from various regions of the UC. However, data from clinical research are mostly obtained using the whole UC or cells from the WJ. The necessity for standardization and adaptation of all methods to GMP conditions is pushing clinical research to create new, safer approaches and cell-based products. Development of cell and tissue cryobanking facilities allows the cord tissue as well as UC-derived cells to be cryogenically saved for further applications. There is a great need for new, highly informative approaches for evaluation of the cells' suitability for clinical administration. However, a balance between thorough scientific research and reasonable implementation of cell-based products into regenerative medicine is required the most.

## Figures and Tables

**Table 1:** Schematic summary of current data on the putative mechanisms of UC-derived cell action.

Objective of therapy	Possible influence	References		
	Direct tissue remodelling (including 2D, 3D biomaterials)	“Type-like” cell effects	Paracrine mechanisms	
Liver		+	+	Campard et al., 2008 [35]; Tsai et al., 2009 [36]; Anzalone et al., 2010 [37]; Shi et al., 2012 [38]; Wang et al., 2013 [39]

Heart and cardiovascular system	+		+	Kadner et al., 2002 [40]; Corrao et al., 2013 [41]; López et al., 2013 [42]; Nascimento et al., 2014 [43]

Kidneys and excretory system			+	Du et al., 2012 [44]; Song et al., 2014 [45]

Central and peripheral nervous systems	+	+	+	Weiss et al., 2003 [46]; Fu et al., 2004 [47]; Fu et al., 2006 [48]; Weiss et al., 2006 [49]; Joyce et al., 2010 [50]; Matsuse et al., 2010 [51]; Carvalho et al., 2011 [52]; Dongmei et al., 2011 [53] Zhang et al., 2011 [54]; Dalous et al., 2012 [55]; Hsieh et al., 2013 [56]; Taran et al., 2014 [57]

Cartilage	+		+	Wang et al., 2009 [58]; Arufe et al., 2011 [59]; lo Iacono et al., 2011 [60]

Metabolic regulation, immune system, and autoimmune diseases			+	Tyndall and Uccelli, 2009 [61]; Mazzini et al., 2010 [62]; Uccelli and Prockop, 2010 [63]; Liu et al., 2014 [64]

Skin	+		+	Shohara et al., 2012 [65]; Arno et al., 2014 [66]

Lungs and respiratory system			+	Lee et al., 2011 [67]; Weiss, 2014 [68]

## Abbreviations

UC: Umbilical cord
MSC: Mesenchymal stem cell
SC: Stem cell
WJ: Wharton's jelly.
